# A closely spaced two-port MIMO antenna with a radiation null for out-of-band suppressions for 5G Sub-6 GHz applications

**DOI:** 10.1371/journal.pone.0306446

**Published:** 2024-07-26

**Authors:** Syed Naheel Raza Rizvi, Md Abu Sufian, Wahaj Abbas Awan, Young Choi, Niamat Hussain, Nam Kim

**Affiliations:** 1 Department of Information and Communication Engineering, Chungbuk National University, Cheongju, South Korea; 2 Department of Intelligent Mechatronics Engineering, Sejong University, Seoul, South Korea; Universiti Brunei Darussalam, BRUNEI DARUSSALAM

## Abstract

This paper presents the design and isolation enhancement of a filtering MIMO antenna with a radiation null for out-of-band suppressions suited for 5G sub-6 GHz communications. The MIMO antenna offers -10 dB impedance bandwidth functionality at the most prominent partial spectrum of the 5G NR n78 band for enabling wireless applications in base stations, ranging from 3.4 GHz to 3.61 GHz. To mitigate the redundancy of an RF filter and to achieve a strong filtering response, a radiation null is produced in the gain with four identical rectangular slots, which results in a significant gain drop of more than 8 dBi at the stopband. The geometrical design also allows 30 percent size reduction of single element. Subsequently, a closely spaced (0.11λ_0_) two-port MIMO antenna is implemented and with the utilization of the proposed rectangular shaped hollow stub parasitic element, the interelement isolation is significantly improved by more than 8 dB over the operational frequency range while retaining the filtering without any additional RF structure. The design simplification, peak gain of 5.4 dBi, near ideal response of diversity gain, ECC less than 0.03, congruency between simulated and measured results, and stable parameters make it a valuable choice for 3.5 GHz sub-6 GHz communications.

## Introduction

A transformation in day-to-day life is projected to be brought about primarily by the development of 5G wireless communications technology [1]. Due to its advantageous innate qualities, it has incorporated several technologies, including automated automation, smart homes, factory automation, and remote healthcare systems [2, 3].

**Fig 1:**
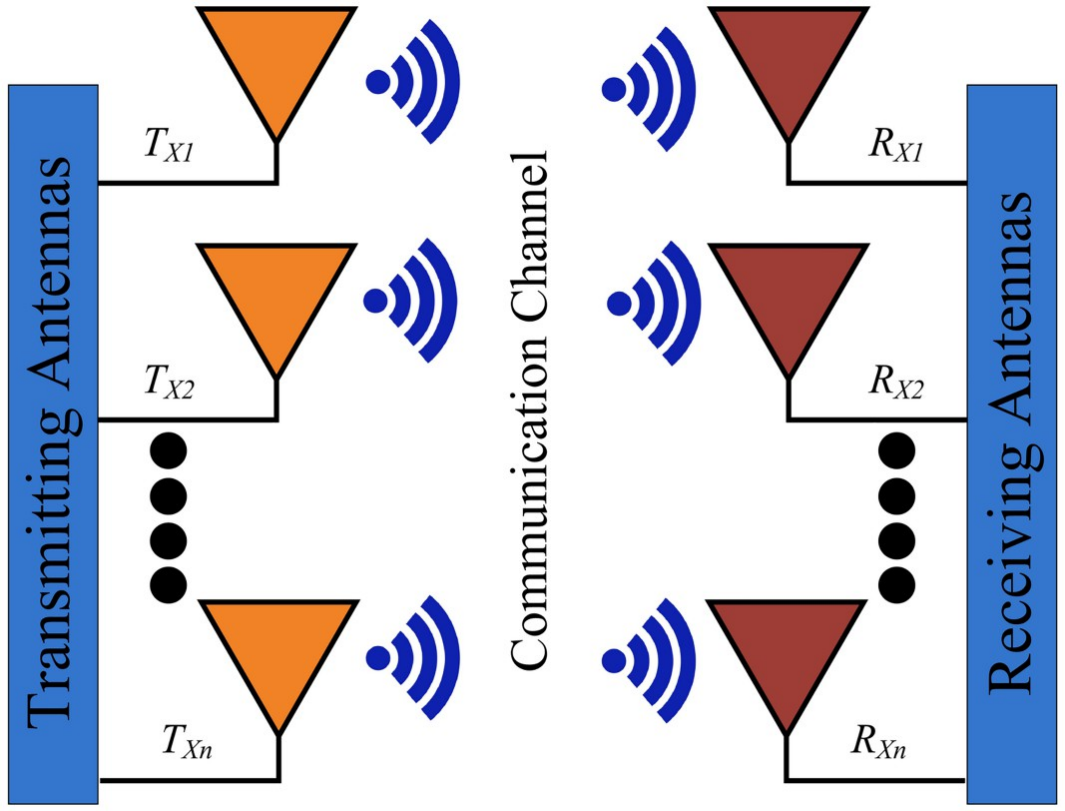
A generic MIMO communication system.

Low latency rates, increased device coverage, energy efficiency, and high data rates are the key features of 5G technology [4–8]. These attributes make 5G an effective standard for futuristic communications. The Federal Communications Commission (FCC) has devised a frequency range for the 5G spectrum, divided into numerous spectrums and their duplex modes to classify the communication standards. Due to the ability of the 5G midband frequency spectrum to carry large amounts of data and its widespread use in society along with interference suppression, it is one of the best choices for wireless communication [9]. The frequency spectrum of 3.4–3.8 GHz, also known as the n78 band, is among the most utilized frequency ranges in the entire world [10]. With the development of more sophisticated technologies, the MIMO (Multiple-Input-MultipleOutput) has emerged as a preferred solution to efficient spectral utilization [11–13]. While maintaining constant power requirements, this approach provides relatively less multipath fading and boosts channel capacity [14–18]. Overall, it enhances radio propagation and spectrum efficiency. Diversity performance, which is appropriate for situations where the connection is between multiple devices, is another significant benefit of MIMO antennas [19, 20]. The fundamental MIMO antenna architecture is depicted in Fig 1. Researchers have used several techniques to increase the efficiency of MIMO systems. Mutual coupling is an essential consideration for effective wireless signal transmission [21]. Thus, thoughtful attempts have been made to mitigate mutual coupling within MIMO systems. In [22], a wideband planer inverted-F MIMO antenna is presented. The design is especially suitable for n77, n78, and n79 band applications. The minimum isolation between the inverted antenna pairs is 10 dB, and the antenna has a 6 dB bandwidth of 3.3 to 7.5 GHz. With a maximum reduction in antenna efficiency of thirty percent when the device is in the user’s hand, an extensive user’s hand impact is investigated in the study.

Transformation electromagnetics (TE) design techniques are the new emerging approaches for isolation enhancement in the antenna elements. The methodology exploited in transformation electromagnetics is to decorrelate the electromagnetic fields, causing a decrease of mutual coupling between the antenna elements. In [23], the researchers present a transformation electromagnetics-based device integrated over the MIMO antenna, allowing mutual coupling reduction between the elements. A dielectric wave tilting structure is applied, which bends the radiating field with a tilt angle of 45 degrees, resulting in a decorrelation between the individual fields of the elements. Furthermore, a defected ground structure (DGS) is used in [24] in a two-element antenna array. The antenna features a combination of metal vias and a T-shaped slot in the antenna ground plane. With an edge-to-edge separation of 49 mm, the device is not suitable for applications with space constraints.

Likewise, a DGS is used in [25] in a three-element antenna array. The antenna features a combination of metal vias and a T-shaped slot in the antenna ground plane. The antenna has close edge-to-edge separation and high isolation between the pairs. However, the bandwidth of the proposed antenna is only 100 MHz, restricting it to low data rates. Moreover, in [26], a dual-polarized MIMO antenna designated to operate at 3.6 GHz frequency for 5G smartphone devices is presented. The proposed design provides a minimum of 12 dB isolation between the antenna elements. The gain for the antenna varies between 4.17 to 6.3 dB which is suitable for high gain applications. However, the overall dimensions of the antenna are large, along with nominal isolation values. A geometrically similar antenna is also proposed in [27], covering a frequency bandwidth of 3.3–3.9 GHz. The envelope correlation coefficient (ECC) is a useful statistic and corresponds to a value of 0.01 in this case. Nevertheless, the antenna’s overall dimensions are considerably greater, corresponding to 150 mm × 75 mm. In [28], researchers present an eight-element MIMO array with a high isolation figure of 17.5 dB. The antenna features moderate radiation efficiency and a low envelope correlation coefficient. Furthermore, the antenna features |*S*_*11*_|< -10 dB impedance bandwidth of 200 MHz. The antenna is geometrically large, has a tolerable ECC value of 0.5, and is suitable for 5G smartphones.

Moreover, a self-isolate MIMO antenna is presented in [29], comprising of a U-shaped stub. A T-shaped stub is etched in the design geometry which allows fine-tuning of the resonance frequency and the impedance matching. Using an extensive parametric analysis, the dimensions of the U-shaped stub are optimized for mutual coupling reduction between the elements. The antenna has a geometrically large size, which may not be suitable for applications where compactness is an important parameter of system design. In [30, 31], the magnitude of the dimensions is large and feature a minimum isolation of 13 dB across the operational range. Some of the isolation enhancement methodologies are listed in Table 1. A multitude of parameters, including feed networks, element configuration, interelement spacing, and filtration integration, impact the isolation. Thus, a trade-off is carried out to maintain equilibrium. These techniques can be extrapolated to isolation enhancement of filtering antennas by balancing impedance matching and retention of filtering characteristics. The research community is eager to design and research on performance enhancement of MIMO antennas operational in the 5G mid-band frequency spectrum with innate filtering abilities. Among all frequency ranges in the globe, the n78 band is one of the most widely used bands for wireless communication applications.

**Table 1:** Isolation enhancement techniques in literature.

Reference.	Isolation Enhancement Technique
[18]	Shorting stub integrated in radiating patch
[19]	45° beam tilt from each other using TE
[20]	Geometric separation between radiating structures
[21]	DGS and metal vias integrated with radiating patch
[22]	0.5 λ_0_ separation between the propagation elements
[23]	Utilization of an L-shaped feedline in the radiating patch

This article presents a filtering MIMO antenna operating at 3.4–3.61 GHz frequency. A radiation null is introduced in the gain of the antenna to retain the filtering characteristics. The antenna has a 5.4 dBi peak gain and offers 8 dB isolation amelioration using a parasitic patch. Compact edge-to-edge separation and filtering capabilities are provided by the architecture without the need for any intricate mutual coupling reduction structure. The design features simple fabrication instructions and is suitable for mass production. The rest of the manuscript is hierarchically organized as follows. The design strategy of the single-unit antenna and its subsequent results are presented in Section 2 (Antenna design configuration and methodology). Furthermore, the MIMO antenna, as well as the mutual coupling reduction phenomenon is explained in subsequent section. The identical section also depicts the hardware prototype and the measurement scenario. The entire discussion is concluded in second last section, along with a comparison with already presented state-of-the-art designs in the literature. For the reader’s convenience, a tabular form is used for comparison of the presented design with the already published designs in the literature, followed by the references afterward.

## Antenna design configuration and methodology

### Single element antenna

**Fig 2:**
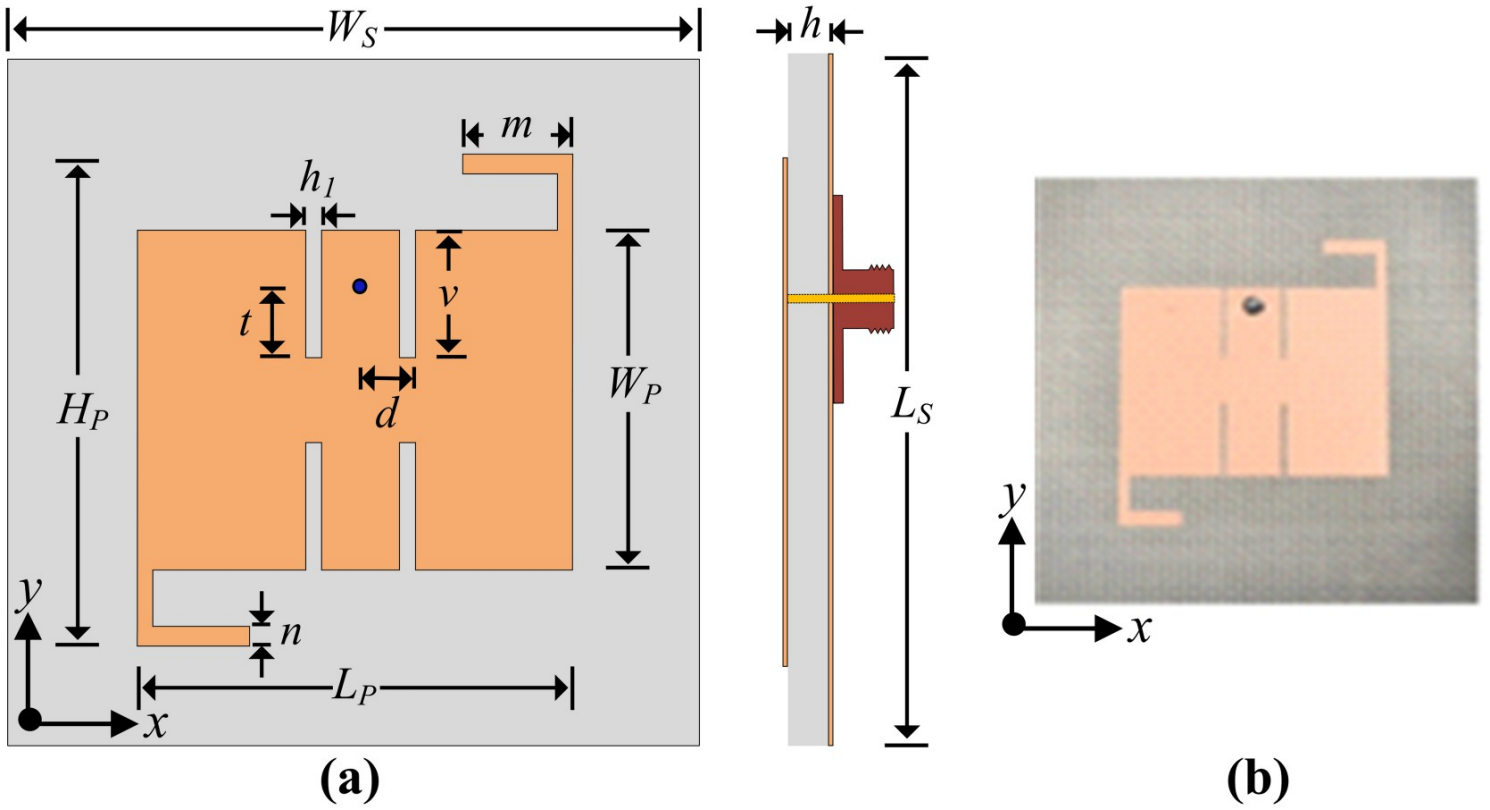
Single-unit antenna (a) proposed geometry and layout, and (b) fabricated design.

Fig 2(a) depicts the top and side viewpoints of the presented antenna. The antenna has compact dimensions of 41.5 mm × 42 mm (*W*_*S*_ × *L*_*S*_), which corresponds to 0.48 λ_0_ × 0.49 λ_0_ in terms of electrical dimensions. The proposed antenna is etched over the commercially available substrate material Rogers/Duroid RO4003C, with a relative permittivity(ε_r_) figure of 3.55, and loss tangent (tanδ) of 0.0027. The material is chosen due to its moderate value of relative permitivity, low loss tangent, and good thermal stability. The proposed radiating patch is fed through a 50 Ω coaxial cable. The geometry of the proposed antenna consists of a rectangular patch with four uniform rectangular slots and a pair of L-shaped stubs at the diagonal ends of the rectangular patch. The design offers suitability for low-cost mass production and mechanical robustness. The optimized parameters of the proposed filtering antenna are as follows: *W*_*S*_ = 41.5; *L*_*S*_ = 42; *H*_*P*_ = 27.05; *h*_*1*_ = 0.46; *v* = 6.82; *L*_*P*_ = 24.25; *W*_*P*_ = 17.8; *d* = 2.99; *h* = 1.524; *t* = 5.75; *n* = 1.23; *m* = 5.6. All mentioned above dimensions are in millimeters (mm).

Typical filtering antennas generally incorporate slot structures [32], parasitic elements [33], metasurfaces [34], defected ground structures [35], and shorting vias [36]. The proposed antenna used a combination of slots and stubs to introduce radiation nulls for out-of-band suppressions. Out-of-band suppressions improve the spectral efficiency and also depreciate the interference between surrounding frequency spectrums. While the design process may involve optimization techniques, the fundamental goal is the suppression of out-of-band frequencies to enhance the antenna’s filtering response.

### Design procedure

Initially, a coaxially-fed rectangular patch antenna is designed with a central frequency of *f*_*r*_ ≈ 3.5 GHz. The coaxial feed line has an impedance bandwidth of 50 Ω. The respective length and the width of the patch can be calculated as [37]:

$$W=\frac{c}{2f_{r}}\sqrt{\frac{2}{{ℇ}_{r}+1}}$$ (1)

$$L=\frac{\text{c}}{2f_{r}\sqrt{{ℇ}_{eff}}}-2\Delta L$$ (2)

Where,

$${ℇ}_{eff}\approx \frac{{ℇ}_{r}+1}{2}+\;\frac{{ℇ}_{r}-\;1}{2}{\left(\;1+12\left(\frac{W_{p}}{H}\right)\right)}^{-0.5}$$ (3)

Where *c* refers to the speed of light in vacuum, ℇ_*r*_ refers to the dielectric constant of the substrate, which is utilized for the design, ℇ_*eff*_ refers to the effective dielectric constant of the patch, and Δ*L* refers to the incremental length of the patch. These equations are predefined antenna design equations essential for understanding and replicating the antenna design process.

**Fig 3:**
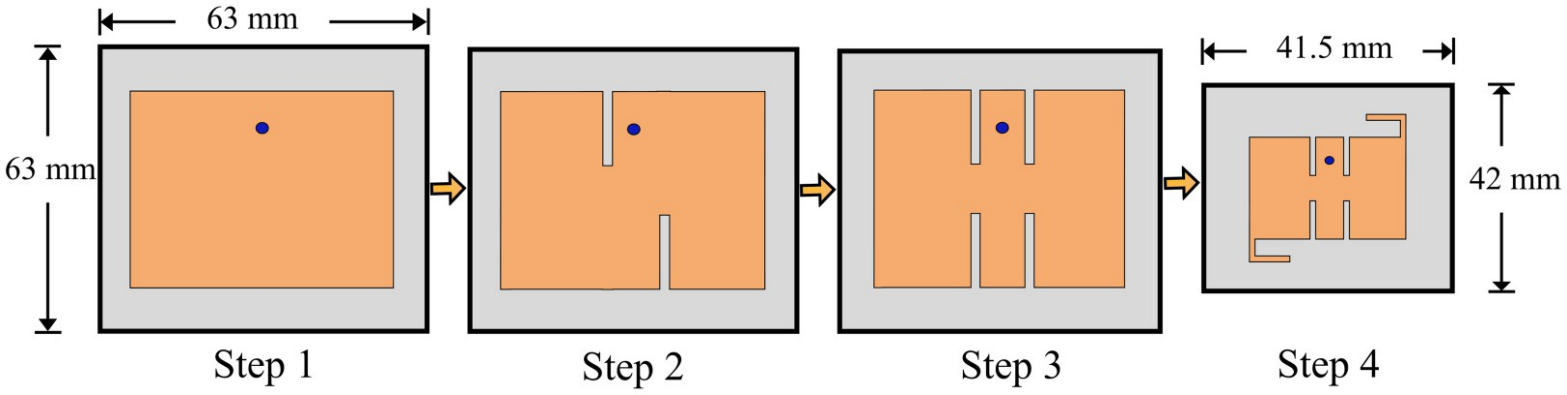
Design evolution of the proposed filtering antenna.

**Fig 4:**
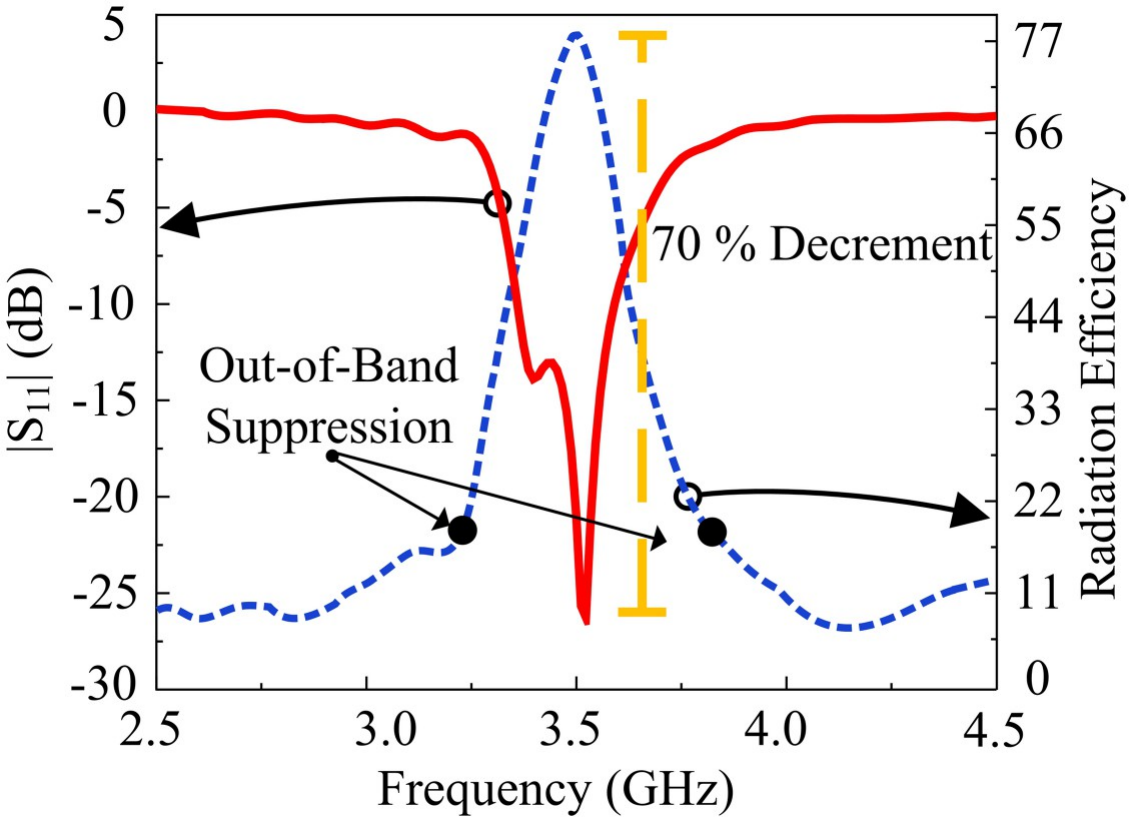
Reflection coefficient and total efficiency of the proposed antenna.

**Fig 5:**
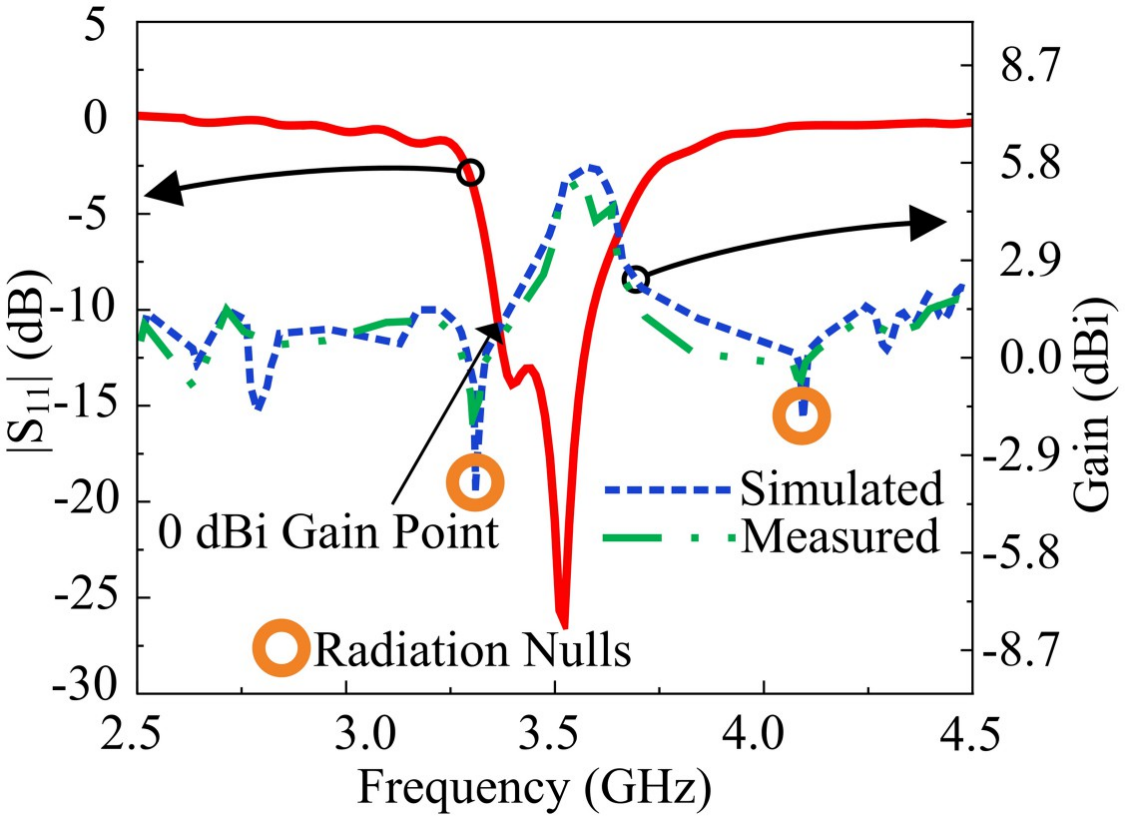
Simulated reflection coefficient and gain (measured and simulated) of the proposed antenna.

Afterwards, two rectangular slots, each of identical length are introduced to the radiating patch. The introduction of rectangular slots introduces a return loss of -20 dB at 3.3 GHz frequency. In the third stage, similar rectangular slots are introduced at the upper and lower end of the rectangular patch, introducing a sharp frequency filtering response from 1 GHz to 5.5 GHz. In the last stage, two L-shaped stubs are introduced at the diagonal corners of the radiating patch, which allows a thirty percent size reduction of the antenna and a higher resonance mode at 3.5 GHz frequency. The proposed antenna has a |*S*_*11*_| < -10 dB impedance bandwidth of 3.4–3.61 GHz. Furthermore, the antenna has an average of |*S*_*11*_| < -3 dB from 1 GHz to 5.5 GHz, with the exception of the operating zone. The filtering characteristics allow attenuation of interference with other frequencies. Thus, the need for an RF filter is mitigated, due to the intrinsic behavior of the antenna to reject other frequency bands. A radiation null at the gain is introduced at the lower stopband which is primarily responsible for the filtering. Fig 3 shows the design steps of the proposed single element. In addition, Fig 4 shows a precipitous drop in overall radiation efficiency, mediated by the radiation null. Fig 5 shows the radiation nulls at specific frequencies.

### Parametric analysis

**Fig 6:**
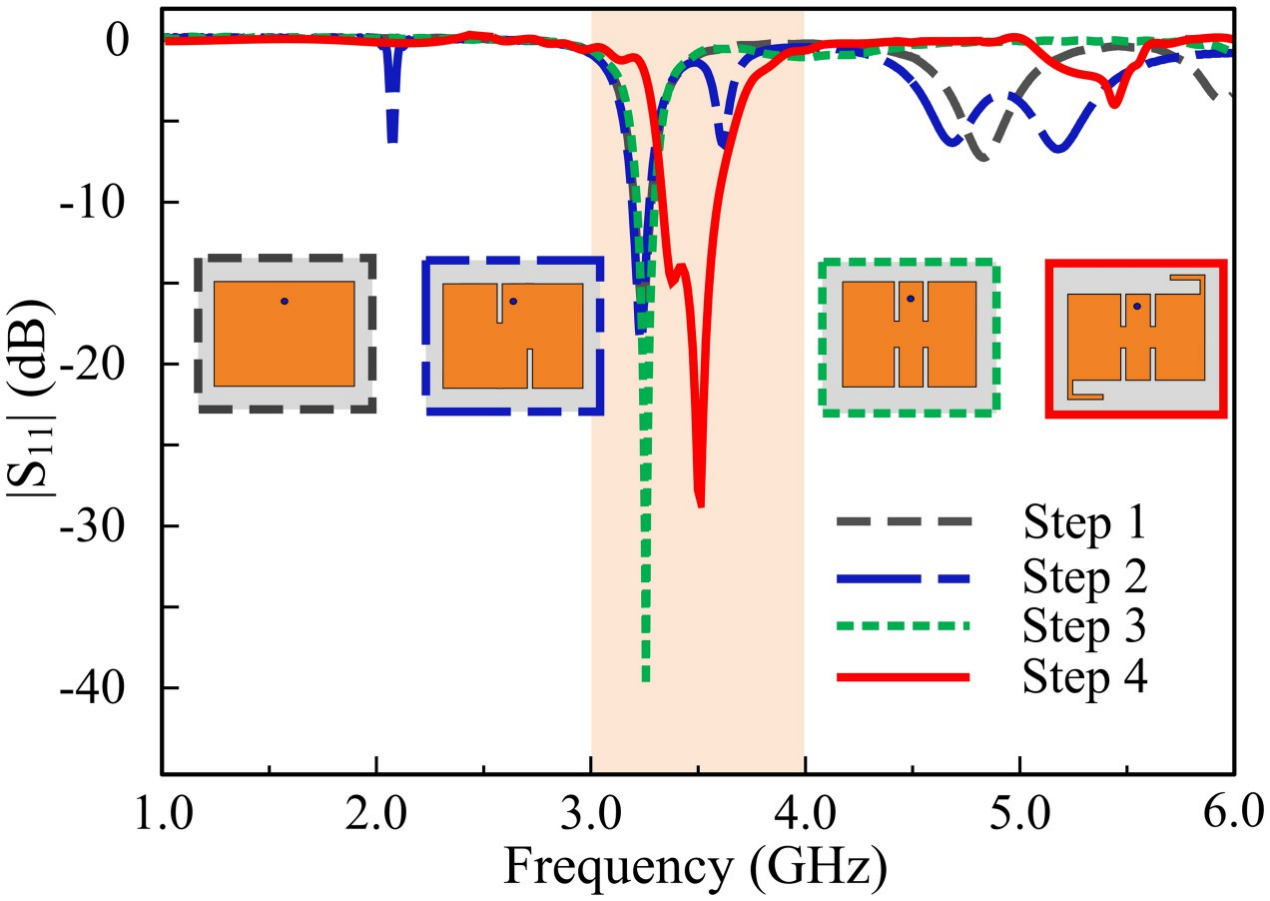
Reflection coefficients at respective stages of the proposed antenna.

A detailed parametric analysis is carried out to illustrate the effects of the various rectangular slots and stubs. The filtering response of the antenna is through the four rectangular slots which cleave the main rectangular patch, whereas the size miniaturization and impedance matching is achieved through the utilization of L-shaped slots at the diagonal ends. Thus, four parameters of the antenna are studied in detail: *m*, *h*_*1*_, *t*, and *v*. Each design stage along with its respective Reflection coefficients are displayed in Fig 6.

The length *v* and width *h*_*1*_ of the rectangular slots are critical to the filtering operation of the antenna. With the increment or decrement in the length of the slot, the antenna shows |*S*_*11*_| < -10 dB impedance bandwidth above 5 GHz frequency. Nearby WLAN-enabled devices can be subject to interference when the antenna has a reflection coefficient value of less than -10 dB above 5 GHz. A similar frequency response is observed when the width of the slots is varied. The antenna displays |*S*_*11*_|< -10 dB impedance bandwidth above 5 GHz frequency with an expansion or shrink in the width of the gap. Therefore, optimal values of the dimensions of the slots are important for the antenna to perform filtering. Nevertheless, the slot dimensions may also be adjusted if the antenna is intended for dual-band operation. As the primary feature in the design is frequency selectivity, we concentrate on the single-band operation in this article.

**Fig 7:**
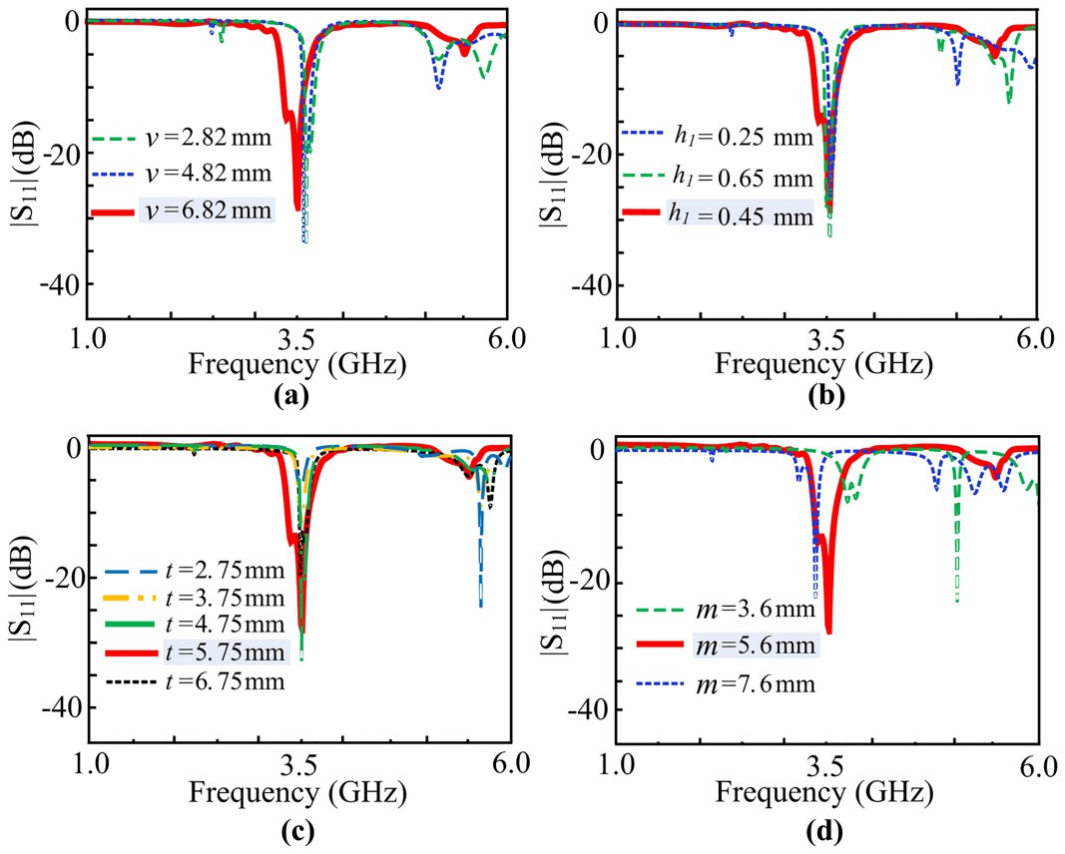
Parametric analysis of the proposed filtering antenna (a) *v* (b) *h*_*1*_ (c) *t*, and (d) *m*.

The position of the feedline, denoted by *t*, as well as the length of the L-shaped stub is an important parameter that shifts the frequency response of the system. As the length of the stub line is incremented, the reflection coefficient |*S*_*11*_| shifts towards the right side of the frequency region. However, with a decrement in the length of the stub line, it is observed that the frequency response shifts towards the left side of the spectrum. To retain filtering capacity and account for the antenna’s dimension requirements, an equilibrium was created between antenna miniaturization and stub length. At 3.75 mm along the vertical axis, the frequency response is shifted towards 3.5 GHz. Consequently, at a 5.75 mm feeding position, maximum impedance matching is achieved at the central frequency of 3.5 GHz. Fig 7 illustrates the parametric analysis of the proposed filtering antenna. Various other dimensional effects are performed to study the effect of length variations. However, the aformentioned parameters are of prime importance. To maintain the brevity of this manuscript, only these four parameters are shown in the parametric analysis stage.

## Results and discussion

### S-Parameters

To validate the simulation results of the presented filtering antenna, we fabricated a hardware prototype and used a Vector Network Analyzer (VNA-Agilent Technologies E8364B) for the measurements of the reflection coefficient. Moreover, the prototype is placed in an electromagnetically shielded environment in an anechoic chamber to measure the far-field parameters of the antenna.

**Fig 8:**
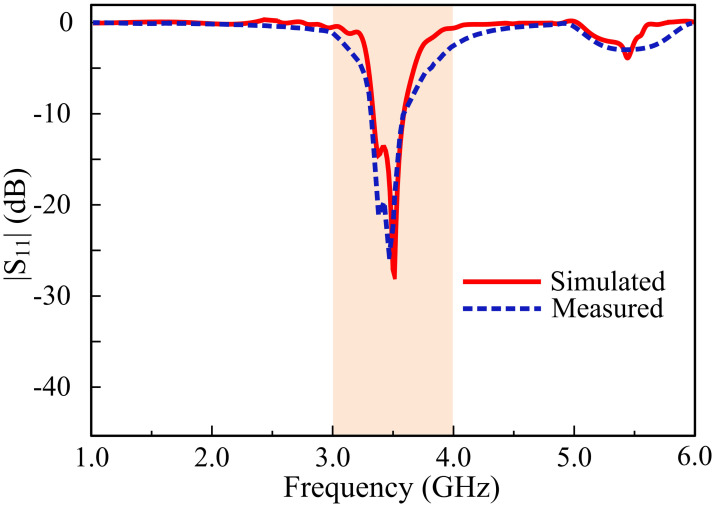
Measured and simulated |*S*_*11*_| response of the proposed antenna.

The simulated and measured reflection coefficient (|*S*_*11*_|) parameters of the highly selective filtering antenna are depicted in Fig 8. The antenna offers a |*S*_*11*_| < -10 dB impedance bandwidth of 3.4–3.61 GHz in simulated results, whereas the measured results indicate |*S*_*11*_| < -10 dB impedance bandwidth of 3.40–3.6 GHz. The measured and simulated results are in conjunction with each other, validating the design of the antenna. Considering *P*_*0*_ be the power output used for the transmission, and *P*_*REF*_ be the reflected power due to the discrepancies between the impedances of the wave generator and the antenna under test, a relation used to describe the reflection coefficient over a specific frequency spectrum is as follows [37].

$$\frac{P_{REF}}{P_{0}}={\left|S_{11\;}\right|}^{2}$$ (4)

### Radiation pattern

The realized antenna gain as a function of spherical coordinates *θ* and *ϕ* is obtained as follows:

$$G_{R}\left(\theta ,\phi \right)=(1-{\left|S_{11}\right|}^{2})G_{A}\left(\theta ,\phi \right)$$ (5)

This can also be expressed as:

$$G_{R}\left(\theta ,\phi \right)=\frac{{\left|S_{21}\left(\theta ,\phi \right)\right|}^{2}}{\left(1-{\left|S_{22}\right|}^{2}\right)}\frac{{\left(4\pi D\right)}^{2}\left(\eta_{REF}\right)}{\lambda^{2}\;\left(G_{R}\right)}$$ (6)

After the simulated and practical measurements of the antenna prototype, it is observed that the antenna offers a peak gain of 5.44 dBi at 3.5 GHz and a stable gain of more than 5.4 dBi throughout the operational region of 3.4–3.61 GHz. The simulated and measured results coincide with each other, thereby making it a favorable candidate for sub-6 GHz applications. The radiation pattern refers to the radio wave emission with respect to the three-dimensional angular coordinates. The power pattern as a function of theta (*θ*) and phi (*ϕ*) angle can be expressed as:

$$P_{R}\left(\theta ,\phi \right)=\frac{{\left|S_{21}\left(\theta ,\phi \right)\right|}^{2}}{\text{max}{\left[S_{21}\left(\theta ,\phi \right)\right]}^{2}}$$ (7)

Whereas the normalized far-field radiation pattern can be expressed as follows [37]:

$$\left|E\left(\theta ,\phi \right)\right|=\frac{S_{21}\left(\theta ,\phi \right)}{\text{max}\left[S_{21}\left(\theta ,\phi \right)\right]}$$ (8)

**Fig 9:**
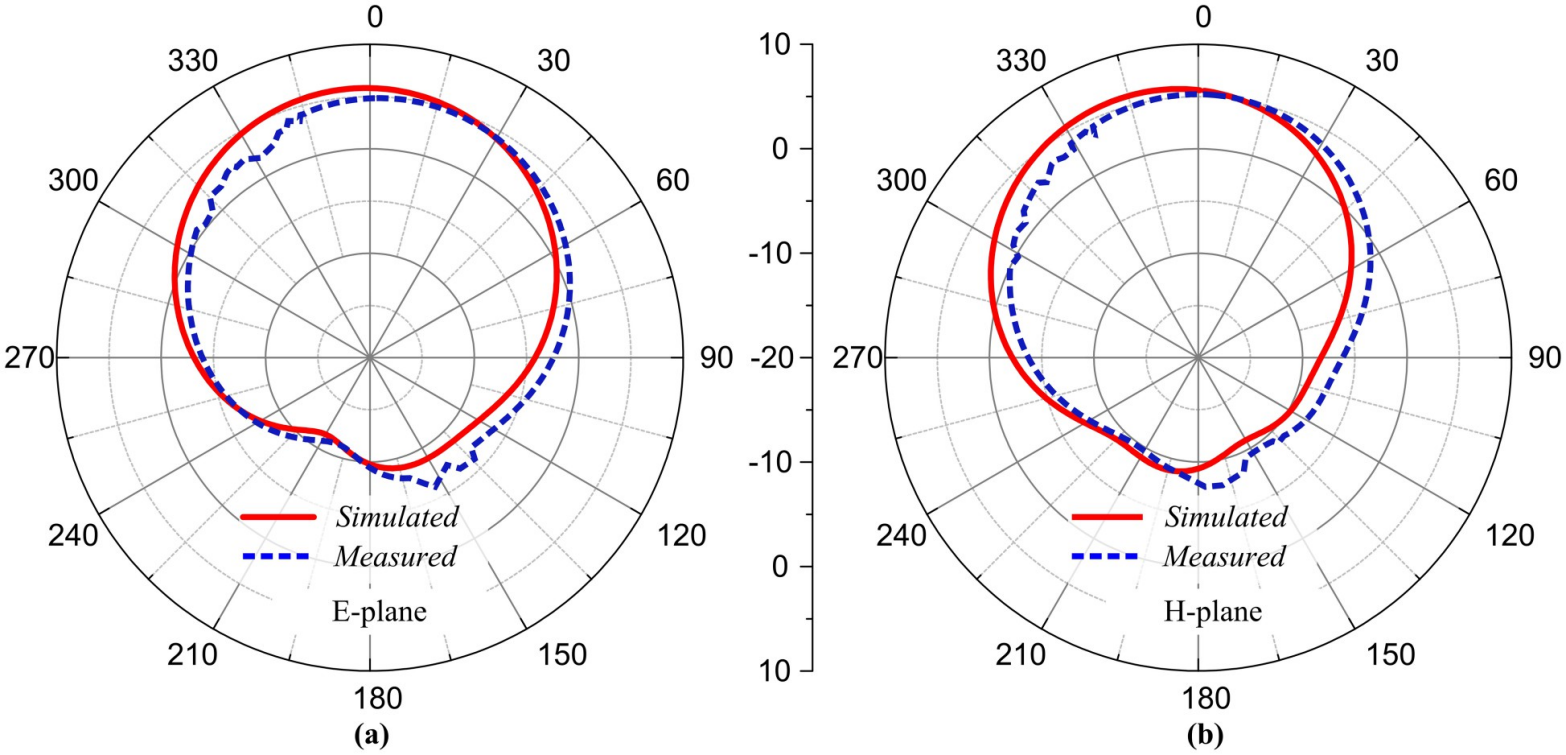
Measured and simulated radiation pattern of the proposed antenna (a) 3.5 GHz at E-plane, and (b) 3.5 GHz at H-plane.

The antenna exhibits a directional radiation pattern due to coaxial feeding. After simulations and measurements, it is suggested that the antenna offers a directional radiation pattern in the E-plane (*θ* = 0°), whereas a similar radiation pattern is observed in the H-plane (*θ* = 90°). Directional radiation pattern allows spectrum efficiency, reduced interference with surrounding bands, increased signal strength in a particular direction, and an overall enhanced range. Fig 9 shows the measured and simulated radiation patterns at 3.5 GHz frequency. The measured and simulated patterns agree with each other.

### MIMO configuration of proposed antenna

**Fig 10:**
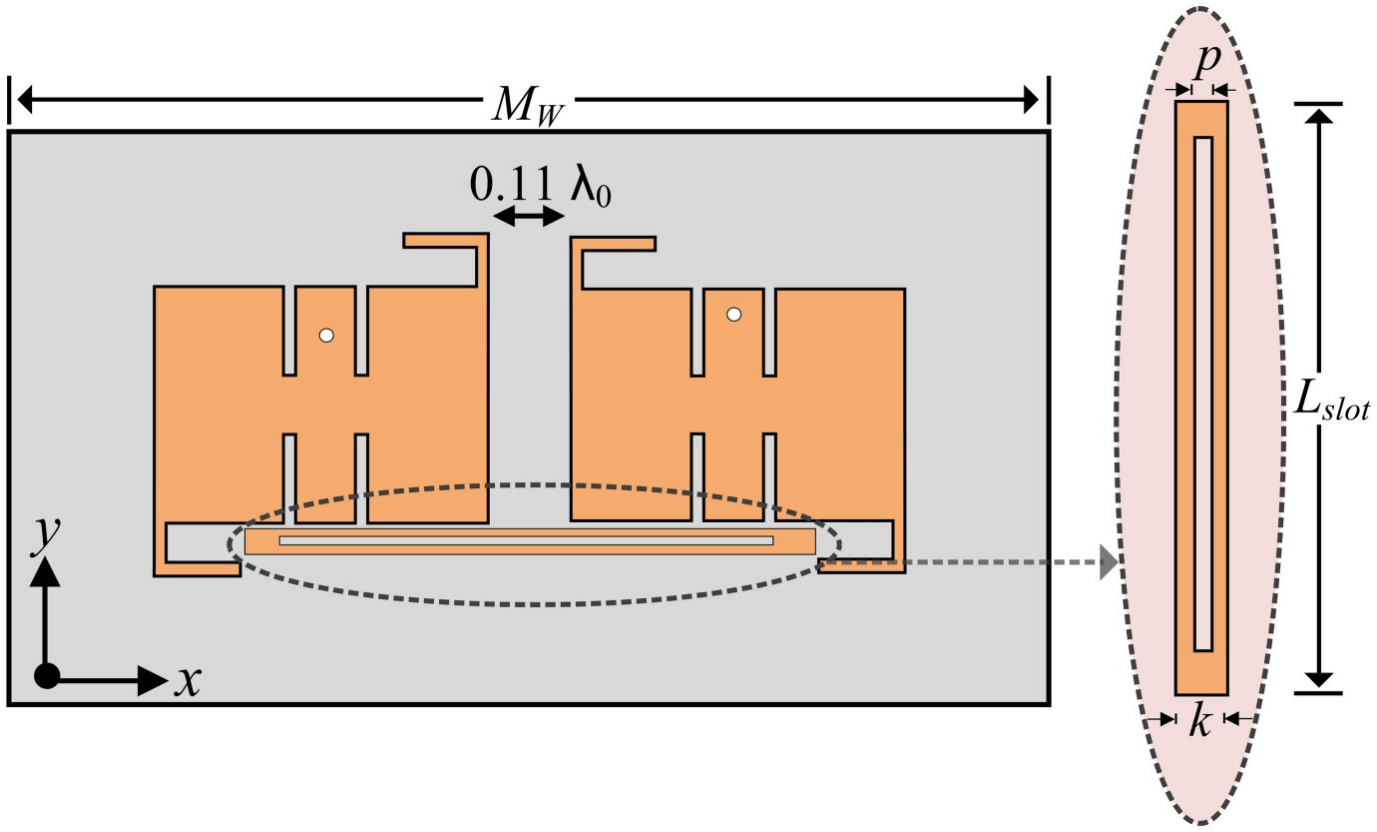
Proposed MIMO antenna and parasitic element loaded in the structure.

The MIMO configuration of the proposed filtering antenna operating at 3.5 GHz frequency is shown in Fig 10. The proposed MIMO antenna consists of two elements on the same axis, with a full ground plane etched on the rear side of the antenna. The MIMO antenna is designed by first flipping the antenna along the vertical axis, and then translating the facsimile of the antenna at the distance of 0.11 λ_0_. Afterward, a hollow stub is added as a parasitic element, which improves the isolation between the antenna pair. A parasitic patch alleviates the amount of power that gets transmitted from one antenna to the adjacent antenna. The transmission characteristics of the antenna demonstrate that the overall isolation is improved with the application of the parasitic patch. The dimensions of the proposed MIMO antenna are as follows: *M*_*W*_ = 73.51 mm, *L*_*slot*_ = 45.05 mm, *p* = 0.45 mm, and *k* = 1 mm.

An explanation of the network model of a MIMO antenna is described in [38, 39]:

$$\left[\begin{array}{c} V_{T}\\ V_{R} \end{array}\right]=\;\left[\begin{array}{cc} Z_{T} & 0\\ H^{OC} & Z_{R} \end{array}\right]\left[\begin{array}{c} i_{T}\\ i_{R} \end{array}\right]$$ (9)

Where *V* and *i* represent the current and voltage vectors, Z represents the impedance matrix, 0 is the zero matrix, and **H**^***OC***^ is MIMO channel matrix. The subscripts “T” and “R” represent the transmitter and receiver parameters. The voltage received which is attenuated by the mutual coupling is as:

$$\left[v\right]=\;Z_{L}\;{\left[Z+Z_{L}\right]}^{-1}v_{oc}$$ (10)

The above-mentioned equation, **v**_**oc**_ represents open circuit voltage, whereas **v** represents voltage received in the presence of mutual coupling. To characterize the spacing between the elements and the number of array components, a simple summation expression is used for evaluating the antenna impact on adjacent elements.

$$MC_{ij}=e^{\left(\frac{-2d_{ij}}{\lambda }\right)\left(\alpha +j\pi \right)}\;;\text{i}\neq \text{j}$$ (11)

$$MC_{ij}=1-\frac{1}{N}\;\sum_{I}\sum_{\;i\;\neq \;j}d_{ij}$$ (12)

Where *d*_*ij*_ represents the gap between the respective *i*^*th*^ and *j*^*th*^ components, *N* is the total count of the components and α represents the fine structure constant.

**Fig 11:**
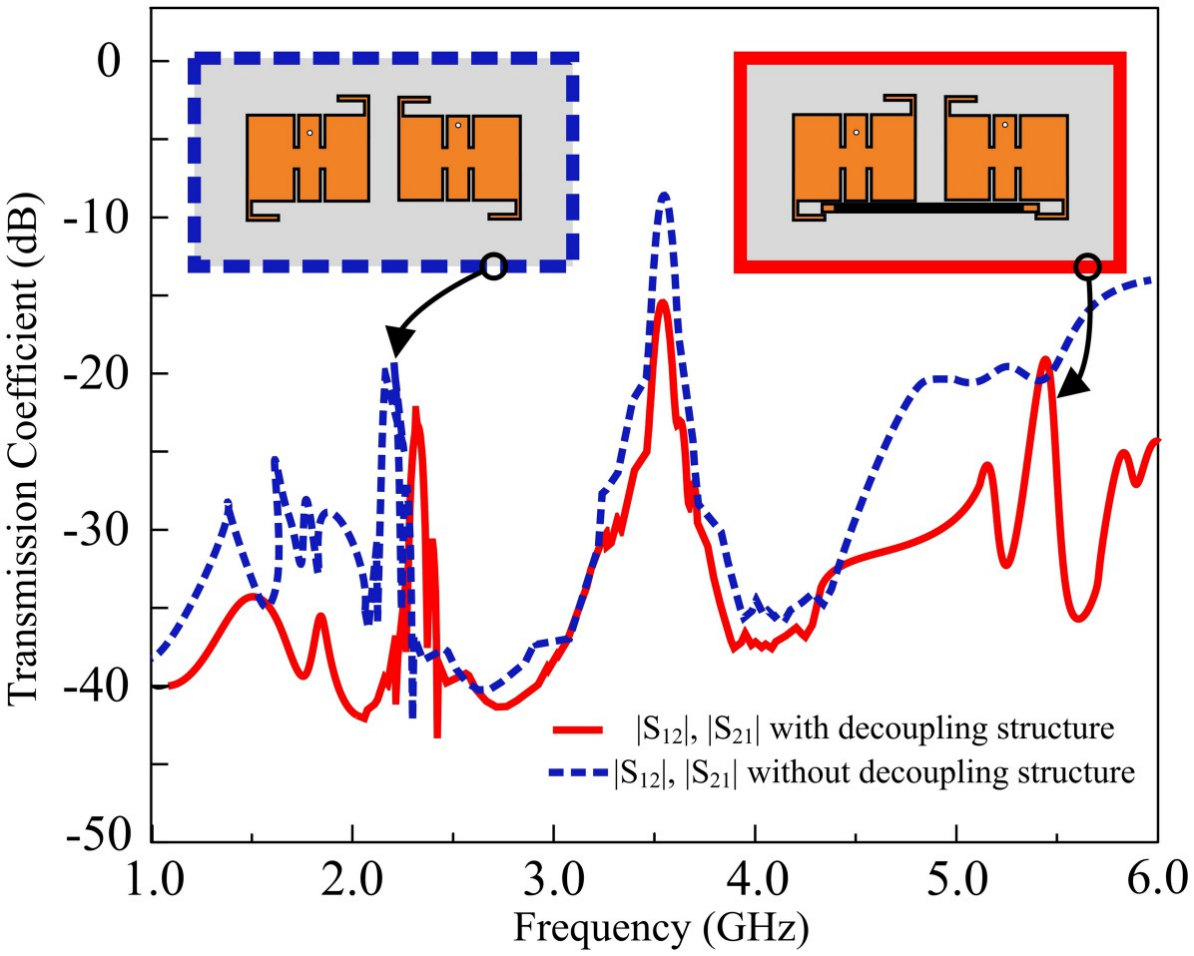
Transmission coefficient with and without the utilization of parasitic patch.

With the addition of a hollow stub at the base of the antennas, the energy transmitted between the elements is depreciated, increasing the minimum value of isolation to 16 dB. Thus, an 8 dB improvement is obtained in isolation by the utilization of a parasitic patch. In the proposed MIMO antenna, various isolation improvement techniques were explored before reaching a compromise between edge-to-edge spacing, filtering characteristics, and isolation amelioration. With the increment in edge-to-edge spacing, the isolation improves, but at the cost of depreciated filtering response by the MIMO antenna. With the inclusion of the DGS, insertion of slots and pins, electromagnetic bandgap structures (EBGs), and parasitic elements between the empty spacing, the filtering of the antenna is also compromised. The frequency response of the transmission parameters of the proposed antenna is displayed in Fig 11.

## Results and discussion

### S-parameters measurements

**Fig 12:**
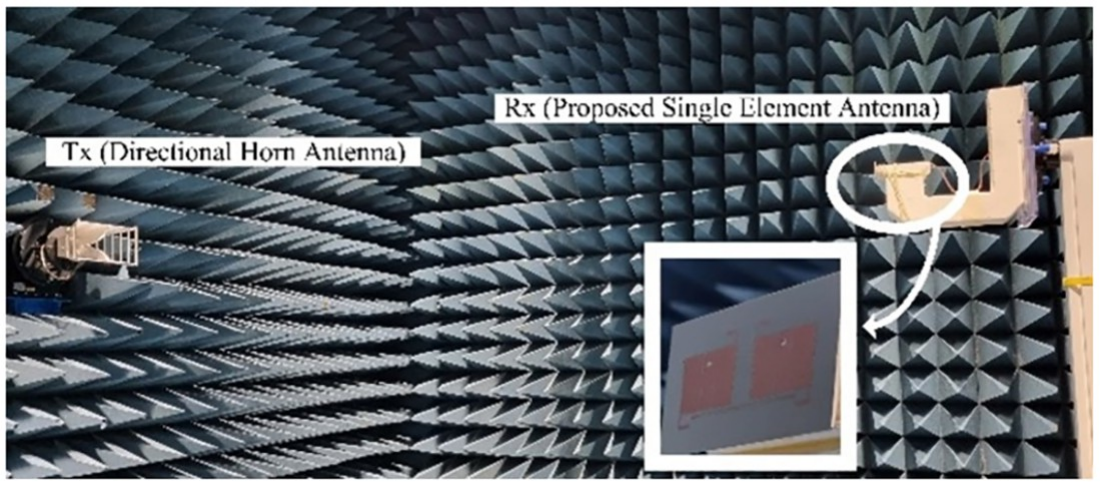
Test antenna in an anechoic chamber for far-field measurements.

The simulations employ commercially available software named CST Microwave Studio. For the practical measurements, a Vector Network Analyzer Vector Network Analyzer (VNA—Agilent Technologies) is used to analyze and verify the design results. Fig 12 illustrates a measurement setup for the proposed MIMO filtering antenna.

**Fig 13:**
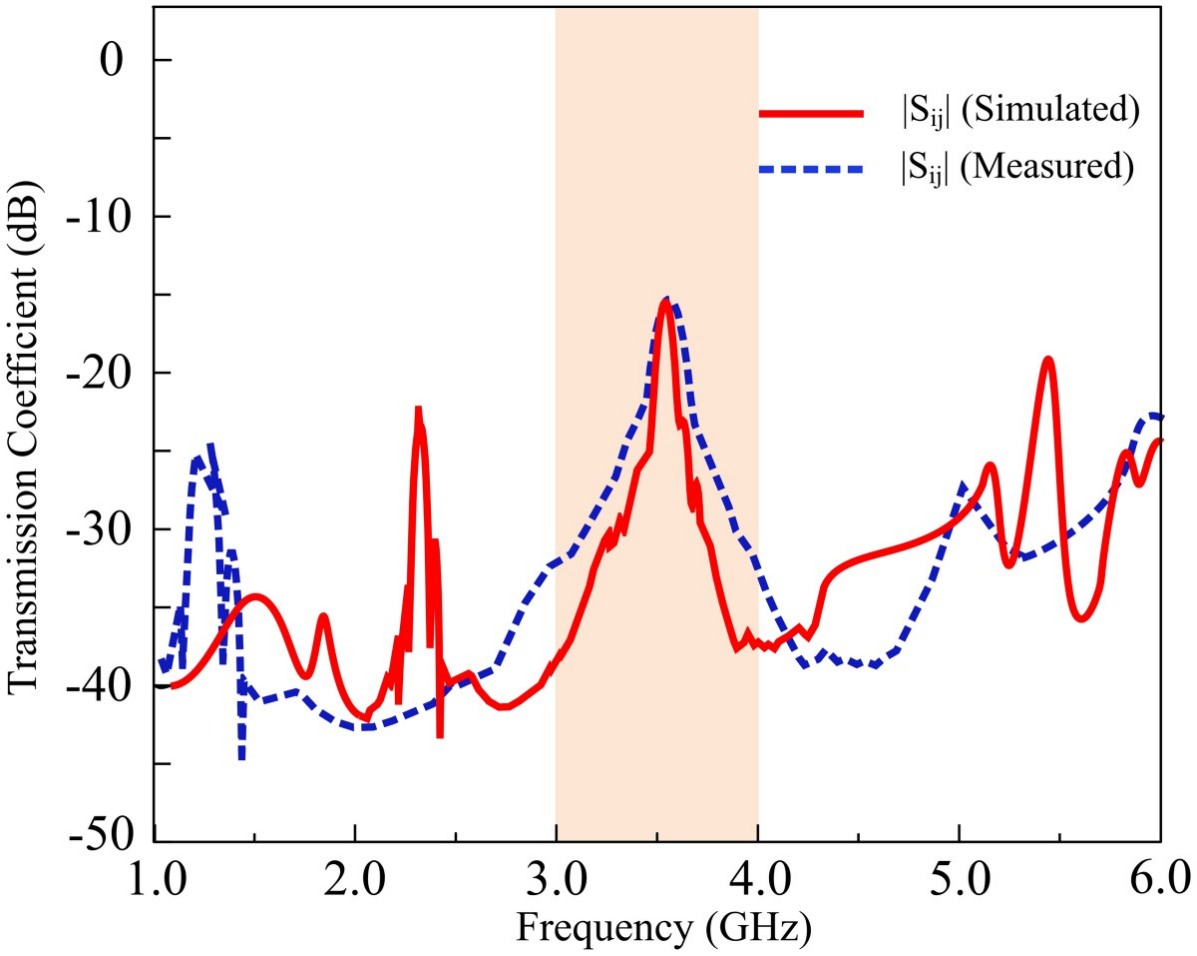
Simulated and measured the transmission coefficient of the proposed antenna.

**Fig 14:**
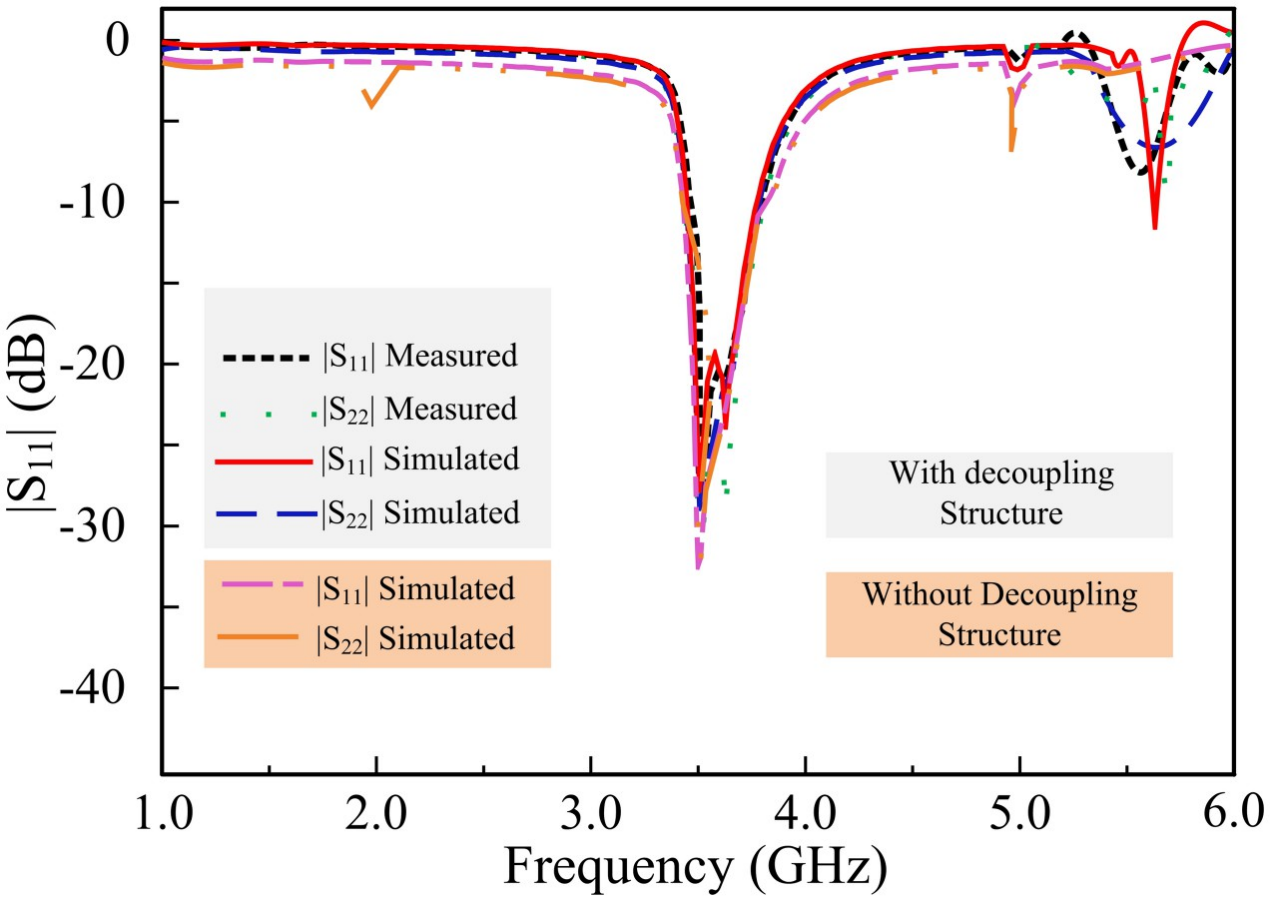
Simulated and measured |*S*_*11*_| and |*S*_*22*_| parameters with and without decoupling structure.

Fig 13 presents the simulated and measured transmission coefficient results of the filtering MIMO antenna. The simulated results indicate that the transmission coefficient value decreased to -16 dB with the inclusion of a parasitic element. Measured findings corroborate the simulated results by demonstrating that the peak transmission coefficient value equates to -16 dB. The energy established between the components is degraded with the insertion of a hollow stub at the base of the antennas, lowering the peak transmission coefficient value, and improving the net isolation between the elements. In addition, Fig 14 shows the simulated and measured |*S*_*11*_| and |*S*_*22*_| parameters with and without decoupling structure.

### Surface current distribution

**Fig 15:**
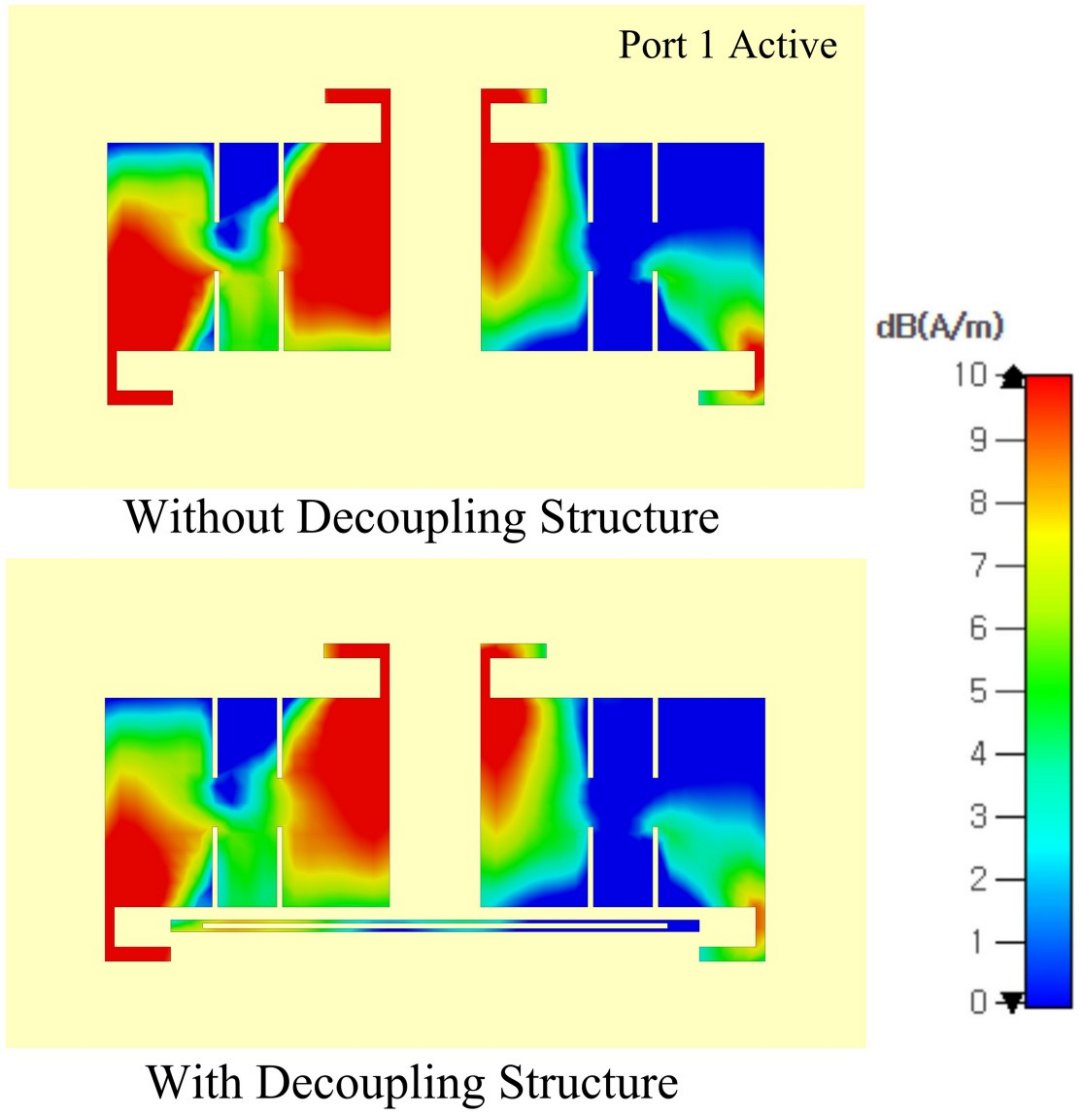
Surface current distribution of the proposed antenna at 3.5 GHz frequency.

The proposed MIMO antenna is excited to analyze the surface current distribution. Fig 15 shows the surface current distribution of the proposed antenna. At 3.5 GHz, the surface current distribution highlights a notable reduction in the flow of current from one antenna element to another. This reduction indicates a decrease in the mutual coupling between adjacent antenna elements, leading to improved isolation and reduced interference between them. The surface current distribution shows in suggested MIMO arrangement, individual components have relatively less effect on the other components when they are active. Due to the hollow nature of the stub etched at the bottom of the MIMO, the current flows between the slot surface, thereby preventing a spread of unwanted currents. As a result, mutual coupling is suppressed.

### Radiation pattern

As stated in the upper section of the article, radiation pattern refers to the radio wave emission with respect to the three-dimensional angular coordinates.

**Fig 16:**
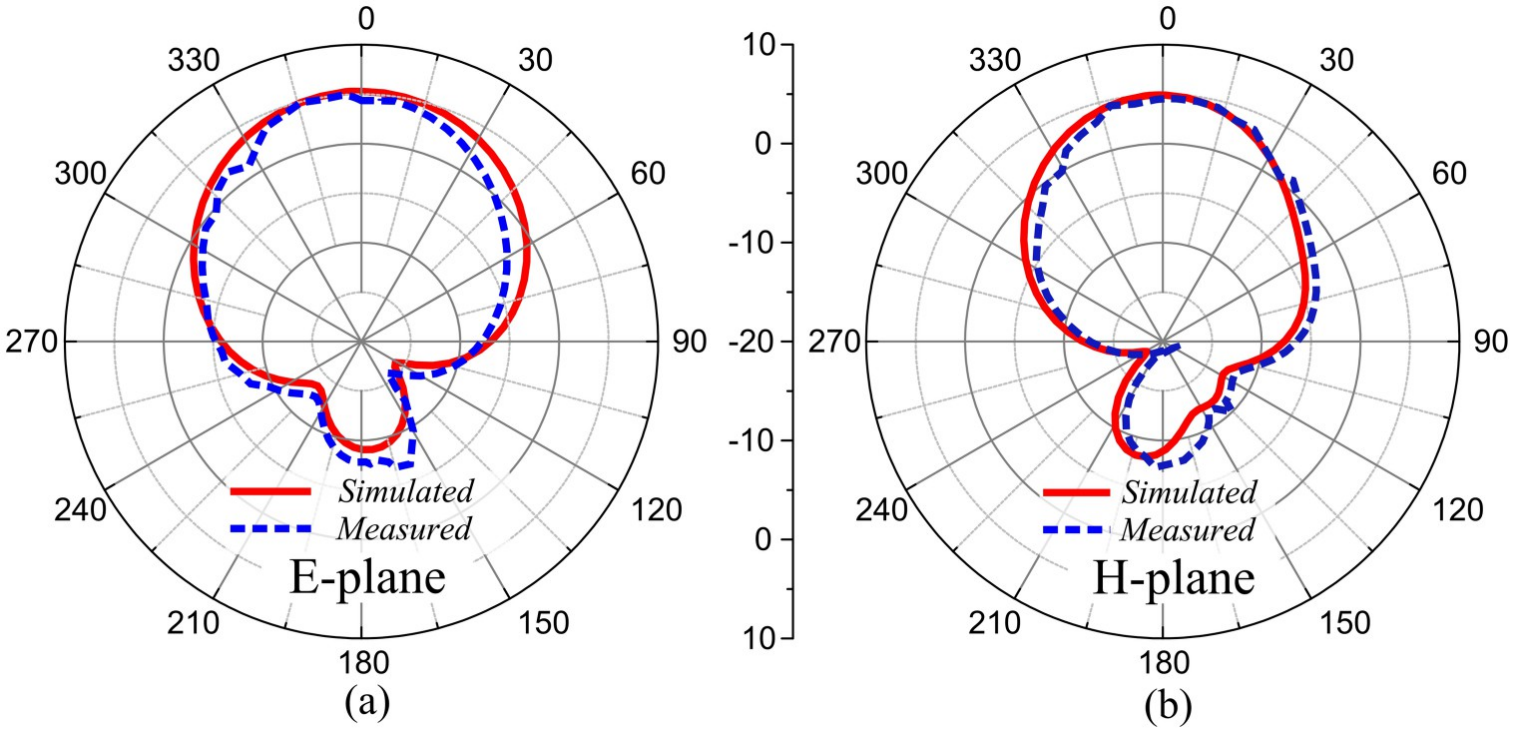
Measured and simulated radiation pattern of the proposed MIMO antenna at 3.5 GHz (a) E-plane, and (b) H-plane.

The antenna exhibits a directional radiation pattern due to coaxial feeding. After simulations and measurements, it is suggested that the antenna offers a directional radiation pattern in the E-plane (*θ* = 0°), whereas a similar radiation pattern is observed in the H-plane (*θ* = 90°). Fig 16 shows the measured and simulated radiation patterns at 3.5 GHz frequency. The measured and simulated patterns agree with each other, which makes the presented antenna a good candidate for wireless applications. The antennas form a geometric symmetry, and their radiation patterns are approximately aligned in their respective planes.

**Fig 17:**
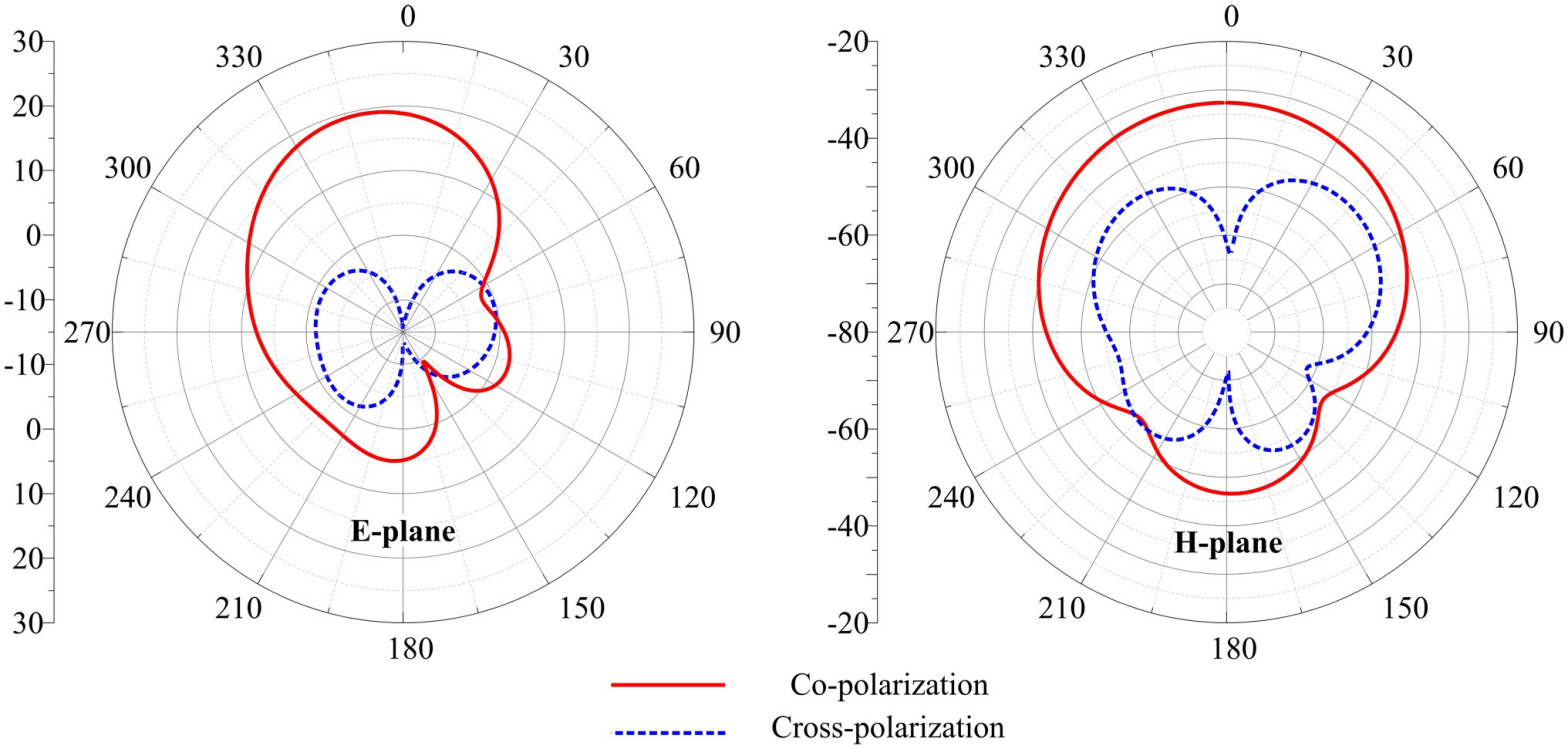
Simulated co/cross polarization of MIMO antenna at 3.5 GHz.

Fig 17 shows the co-polarization and cross-polarizations 2D patterns of the proposed antenna at E and H plane. The minimum acceptable gap between co/cross-polarization of around 15 dB is obtained, which enables adequate pattern diversity.

### Envelope correlation coefficient and diversity gain

The Envelope Correlation Coefficient (ECC) is a metric for determining how independently each antenna radiates. It is a measurement of the degree to which the radiation patterns of the elements of the MIMO antenna are correlated. The ideal value of ECC corresponds to zero, with the maximum acceptable value of 0.5 is tolerated. The proposed MIMO antenna has an ECC value of less than 0.03. ECC can be found by the following equation [40]:

$$\rho_{eij}=\;\frac{{\left|{S_{ii}}^{*}\;S_{ij}+{S_{ji}}^{*}\;S_{jj}\right|}^{2}}{\left(1-|S_{ii}{|}^{2}-{S_{ij}}^{2}\right)\left(1-|S_{ji}{|}^{2}-{S_{jj}}^{2}\right)}$$ (13)

Alternatively, ECC can also be calculated using complex radiation pattern. For *N* element MIMO antenna, we can use hermition operator over [***F***_***i***(***θ***,***ϕ***)_] and [***F***_***j***(***θ***,***ϕ***)_] complex radiation fields to calculate the value of ECC.


$$\rho_{c\left(i,\;j,\;N\right)}=\;\frac{\iint [F_{i\left(\theta ,\;\phi \right)}.F_{j\left(\theta ,\;\phi \right)}]d\left(\theta ,\;\phi \right)}{\sqrt{\iint |F_{i\left(\theta ,\;\phi \right)}{|}^{2}d\left(\theta ,\;\phi \right)\iint |F_{j\left(\theta ,\;\phi \right)}{|}^{2}d\left(\theta ,\;\phi \right)]}}$$ (14)

**Fig 18:**
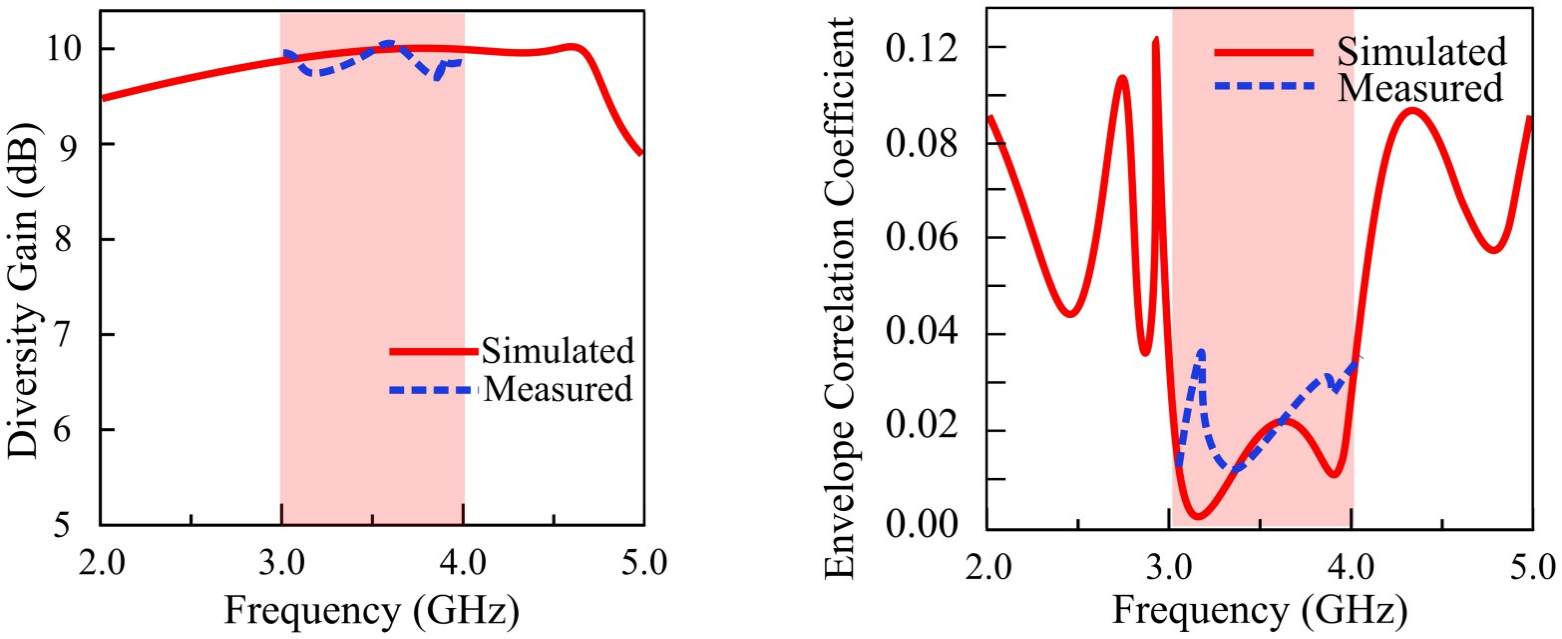
ECC and DG of the proposed MIMO antenna.

Another measure for analyzing the transmission power losses that occur when a diversity scheme is implemented is called Diversity Gain (DG). The ideal value of DG equates to 10 dB. The proposed antenna has a DG value of 9.99 dB, close to the ideal value of 10 dB. Fig 18 shows the DG and ECC of the antenna in the operational bandwidth. Mathematically, DG can be evaluated as follows [41]:

$$DG=\;\sqrt{1-{\left|ECC\right|}^{2}}$$ (15)

### Mean effective gain (MEG) and channel capacity loss (CCL)

**Fig 19:**
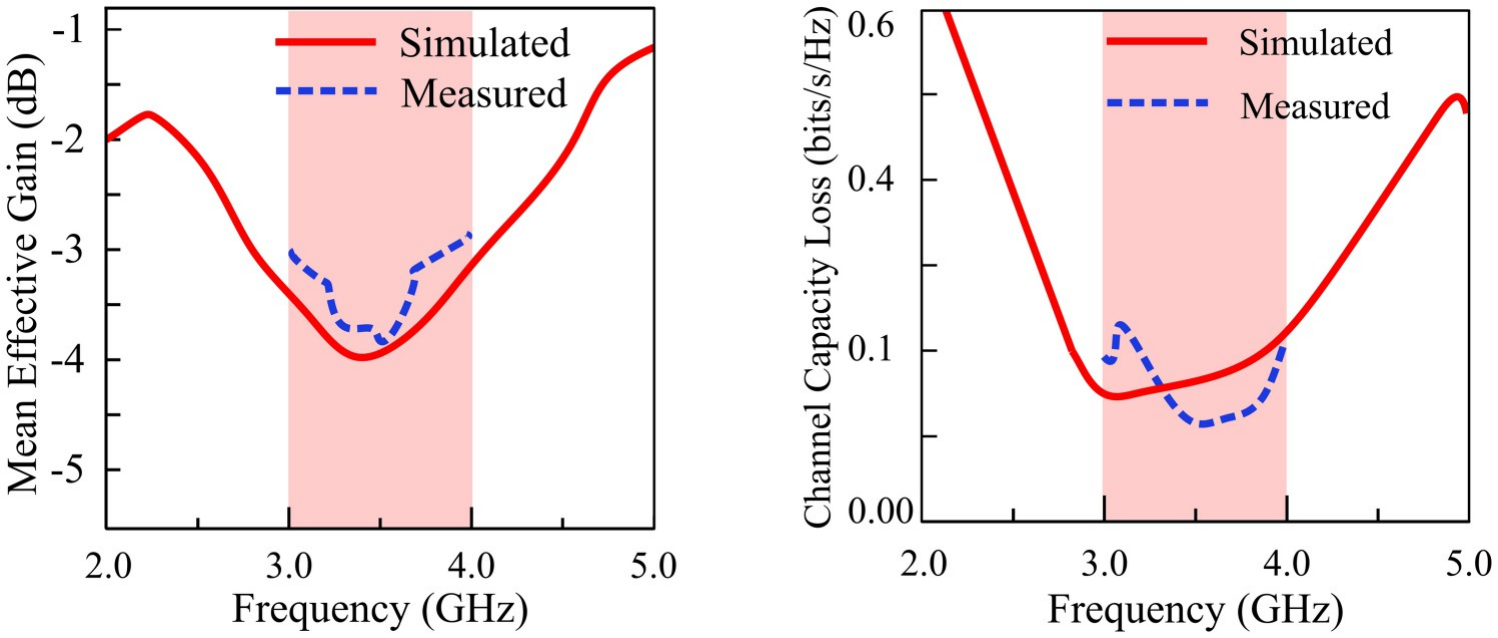
MEG and CCL of the proposed MIMO antenna.

Information may be reliably carried via a communication channel at a maximum rate, which is referred to as channel capacity. Fig 19 shows the CCL and MEG results of the proposed filtering MIMO antenna. For the practical cases, CCL cannot exceed 0.4 bps/Hz. The CCL may be calculated as:

$$CCL=\;-log_{2}det\left|\psi^{R}\right|$$ (16)

Where *ψ*^*R*^ refers to:

$$\left|\psi^{R}\right|=\;\left[\begin{array}{cc} \rho_{11} & \rho_{12}\\ \rho_{21} & \rho_{22} \end{array}\right]$$ (17)

Mean Effective Gain (MEG) is the ratio of mean received power in a fading environment to the mean incident power. For the practical cases, MEG should be below the threshold value of <-3 dBi. The MEG can be evaluated as:

$$MEG=0.5\;\left[1-\sum_{i,j=1}^{n}\left|S_{ij}\right|\right]$$ (18)

### Performance comparison

This subsection outlines the performance comparison with already published methodologies and state-of-the-art methods. Table 2 gives a comprehensive overview of the performance indicators of the presented MIMO antenna and allows for evaluation against previously published research in the literature.

**Table 2:** Performance evaluation of the proposed MIMO antenna with previous literature.

Ref	Antenna Design Methodology	Dimensions (mm ×mm)	Operating Band (GHz)	Minimum Inter-element Spacing (With Respect to Central Frequency)	Filtering Response	Minimum Isolation (dB)	ECC
[22]	Planar Inverted-F MIMO Antenna	150 × 75	3.3–7.7 (6-dB)	0.33 λ	No	≥ 10	< 0.15
[24]	Shared Aperture MIMO Antenna	120 × 60	1.82–2.14 1.4–1.58 4–4.5 3.1–3.8	0.24 λ	No	≥ 15	< 0.15
[25]	MIMO Patch Antenna using DGS	76.6 × 44	3.94–4.06	0.037 λ	No	≥ 22.9	Not reported
[27]	Ring Slot Element-Based MIMO Antenna	150 × 75	3.3–3.9	0.33 λ	No	≥ 17	< 0.01
[28]	Open Slot Element-Based MIMO	150 × 80	3.4–3.6	0.22 λ	No	≥ 17.5	< 0.15
[29]	U-Shaped Patch Element	150 × 75	3.4–3.6	0.24 λ	No	≥ 19	< 0.01
[31]	Coupled Loop MIMO Antenna	150 × 70	0.69–0.96 1.71–2.69	0.05 λ	No	≥ 10	< 0.5
[42]	Symmetrical Slots MIMO Antenna	155 × 75	3.4–3.6 4.8–4.9	≈0.2 λ	Yes (Filter integrated)	≥ 11.8	< 0.1
[43]	DGS-Based Monopole MIMO	60 × 40.5	3–3.5	0.25 λ	Yes	≥ 18.5	< 0.015
[44]	Rectangular Iterated Geometry-Based MIMO	31.5 × 45	3.1–4.7 5.6–8.5	0.2 λ	Yes	≥ 17	< 0.009
[45]	Loop Type Radiator Based MIMO	150 × 75	2.38–2.72 3.19–3.84	0.148 λ	No	≥ 15	<0.17
[46]	Slot Integrated Planer Antenna	30 × 40	3.2–5.85	0.12 λ	No	≥ 17	< 0.01
[47]	Monopole Radiator with Dollar Shaped Decoupling structure	45 × 30	2.4–2.57 3.85–6.96	0.04 λ	No	≥ 15	< 0.02
[48]	Transparent Antenna with Partial Ground Plane	66 × 45	2.2–6	0.15	No	≥ 15	< 0.016
**This Work**	**Monopole Patch with Parasitic Element**	**73.5 × 42**	**3.4–3.6**	**0.11 λ**	**Yes**	**≥ 16**	**< 0.03**

In [20, 22, 25, 37], the operational bandwidth of the proposed MIMO antennas is more than the bandwidth of the presented antenna in this literature. Nonetheless, the edge-to-edge distance between the elements is above 0.2 λ, which is twice as far apart as the antenna suggested. Additionally, the layout geometry of the antennas also demands more space. Furthermore, the antennas do not exhibit the filtering response, thereby subject to interference with nearby WLAN-enabled devices. In [40], a geometrically complex MIMO antenna is presented with filtering response. The design necessitates intricate production procedures since the RF filter is incorporated into the structure. As the RF filter is designed using electrical components, they are prone to increase the design costs as well. Moreover, the edge-to-edge separation is also twice the distance in this proposed MIMO antenna.

Similarly, in [37], a high-isolation MIMO antenna is presented with geometrically simple architecture. However, the spacing between the elements is about 0.25 λ, not suitable for applications which impose strict minimum edge-to-edge separation. A ring-slot element-based MIMO antenna with a frequency bandwidth of 3.3 to 3.9 GHz is presented as well in [25]. The ECC in this design equals a value of 0.01. Nevertheless, the antenna’s overall dimensions are considerably greater, corresponding to 150 mm × 75 mm and the edge-to-edge separation above the value of 0.25 λ. In [26], researchers present an eight-element MIMO array with a high isolation figure of 17.5 dB. The antenna features moderate radiation efficiency and a low envelope correlation coefficient. Furthermore, the antenna features |*S*_*11*_|< -10dB impedance bandwidth of 200 MHz. The antenna is geometrically larger compared to the antenna presented in this article. Moreover, a self-isolated MIMO antenna is presented in [27], comprising of a U-shaped stub. A T-shaped stub is also included in the design geometry which allows fine-tuning of the resonance frequency and the impedance matching. The antenna has a geometrically large size, which may not be suitable for applications where compactness between separation is an important requirement. Our proposed filtering MIMO antenna has a compact dimension, a geometrically simple layout, a compact spacing of 0.11 λ, an ECC value of < 0.03 across the operating range, a sharp filtering response to mitigate interference with nearby ISM and WLAN (802.11j/y/a/h/n/ac) bands, along with a peak gain of 5.4 dB suitable for 5G sub-6 GHz wireless applications. Reduced interelement spacing enables a denser antenna configuration, which enhances spatial multiplexing and improves the overall performance of MIMO systems, particularly in densely populated or interference-prone environments.

## Conclusion

This paper describes a filtering MIMO antenna for 5G sub-6 GHz communications. The proposed design features compact dimensions, geometrically straightforward architecture, an Envelope Correlation Coefficient (ECC) value of < 0.03 over the operational range, and isolation amelioration of 8 dB while maintaining the geometrical spacing below 0.12λ. The volume of the single element is decreased by inserting two L-shaped stubs at the diagonal corners of the radiating patch. The two-unit MIMO antenna is designed by first flipping the facsimile of a single antenna along vertical axis and then translating it along the x-axis. With the aid of a parasitic component, 8 dB isolation is ameliorated, bringing the minimum isolation value to 16 dB across the operational bandwidth of the proposed MIMO antenna. There is an agreement between the measured and simulated results of the MIMO antenna. The antenna has a nearly optimal diversity gain of 9.99, a Channel Capacity Loss (CCL) of less than 0.4 bps/Hz, and a Mean Effective Gain (MEG) value of less than -3 dB throughout the operational region. The antenna undergoes comparison with the novel designs found in previous works. The proposed MIMO antenna is a viable option for 3.5 GHz sub-6 GHz applications owing to its simple model and miniature design, filtering across various frequency bands, and stable characteristics.
